# Kava for the treatment of generalised anxiety disorder (K-GAD): study protocol for a randomised controlled trial

**DOI:** 10.1186/s13063-015-0986-5

**Published:** 2015-11-02

**Authors:** Karen M. Savage, Con K. Stough, Gerard J. Byrne, Andrew Scholey, Chad Bousman, Jenifer Murphy, Patricia Macdonald, Chao Suo, Matthew Hughes, Stuart Thomas, Rolf Teschke, Chengguo Xing, Jerome Sarris

**Affiliations:** The University of Melbourne, Department of Psychiatry, The Melbourne Clinic, Melbourne, Australia; Swinburne University of Technology, Centre for Human Psychopharmacology, Swinburne, Australia; Department of Psychiatry, The University of Queensland, Melbourne, Australia; Department of Psychiatry, The University of Melbourne, Parkville, Australia; Florey Institute of Neuroscience and Mental Health, The University of Melbourne, Parkville, Australia; Department of General Practice, The University of Melbourne, Parkville, Australia; Brain and Mental Health Laboratory, School of Psychological Science, Monash University, Monash, Australia; Swinburne University of Technology, Brain and Psychological Sciences Centre, Swinburne, Australia; School of Psychological Science, Monash University, Monash, Australia; Department of Internal Medicine II, Section of Gastroenterology and Hepatology, Klinikum Hanau, Teaching Hospital of the Johann Wolfgang Goethe University of Frankfurt/Main, Frankfurt, Germany; Department of Medicinal Chemistry, College of Pharmacy, University of Minnesota, Duluth, USA

**Keywords:** Protocol, Anxiety, GAD, RCT, Kava, Kavalactones, GABA, Anxiolytic, Nutraceutical

## Abstract

**Background:**

Generalised anxiety disorder (GAD) is a chronic and pervasive condition that generates high levels of psychological stress, and it is difficult to treat in the long term. Current pharmacotherapeutic options for GAD are in some cases only modestly effective, and may elicit undesirable side effects. Through targeted actions on the gamma-aminobutyric acid (GABA) pathway, the South Pacific medicinal plant kava (*Piper methysticum*) is a non-addictive, non-hypnotic anxiolytic with the potential to treat GAD. The evidence for the efficacy of kava for treating anxiety has been affirmed through clinical trials and meta-analyses. Recent research has also served to lessen safety concerns regarding the use of kava due to hepatotoxic risk, which is reflected in a recent German court overturning the previous kava ban in that country (which may in turn influence a reinstatement by the European Union). The aim of current research is to assess the efficacy of an ‘aqueous noble cultivar rootstock extract’ of kava in GAD in a larger longer term study. In addition, we plan to investigate the pharmacogenomic influence of GABA transporters on response, effects of kava on gene expression, and for the first time, the neurobiological correlates of treatment response via functional and metabolic imaging.

**Methods/Design:**

This clinical trial is funded by the Australian National Health and Medical Research Council (APP1063383) and co-funded by MediHerb (Integria Healthcare (Australia) Pty. Ltd). The study is a phase III, multi-site, two-arm, 18-week, randomised, double-blind, placebo-controlled study using an aqueous extract of noble kava cultivar (standardised to 240 mg of kavalactones per day) versus matching placebo in 210 currently anxious participants with diagnosed GAD who are non-medicated. The study takes place at two sites: the Centre for Human Psychopharmacology (Swinburne University of Technology), Hawthorn, Melbourne, Australia; and the Academic Discipline of Psychiatry (The University of Queensland) based at the Royal Brisbane and Women’s Hospital, Herston, Brisbane, Australia. Written informed consent will be obtained from each participant prior to commencement in the study. The primary outcome is the Structured Interview Guide for the Hamilton Anxiety Rating Scale (SIGH-A). The secondary outcomes involve a range of scales that assess affective disorder symptoms and quality of life outcomes, in addition to the study of mediating biomarkers of response (assessed via genomics and neuroimaging).

**Discussion:**

If this study demonstrates positive findings in support of the superiority of kava over placebo in the treatment of GAD, and also is shown to be safe, then this plant-medicine can be considered a ’first-line‘ therapy for GAD. Genomic and neuroimaging data may reveal clinical response patterns and provide more evidence of the neurobiological activity of the plant extract.

**Trial Registration Information:**

ClinicalTrials.gov: NCT02219880 Date: 13 August 2014:

## Background

Generalised anxiety disorder (GAD) is defined through cognitive and somatic symptomatology; involving primarily excessive chronic worry and anticipatory anxiety [1], in addition to a constellation of presentations such as sleep disturbances, restlessness, edginess/irritability, fatigue, concentration difficulties, and muscular tension [2]. GAD is ubiquitous and persistent, usually with early onset and high prevalence of co-morbid affective disorders and substance misuse [3–6]. Unfortunately, available pharmacotherapies have a modest clinical effect [7, 8] and may elicit undesirable side effects [9–11], and while psychotherapeutic treatments are found to be efficacious [12, 13], they are of limited utility for some patients [14, 15]. As a result, many GAD sufferers do not receive adequate treatment [16–18], thereby providing an impetus to explore other therapeutic options.

**Fig. 1:**
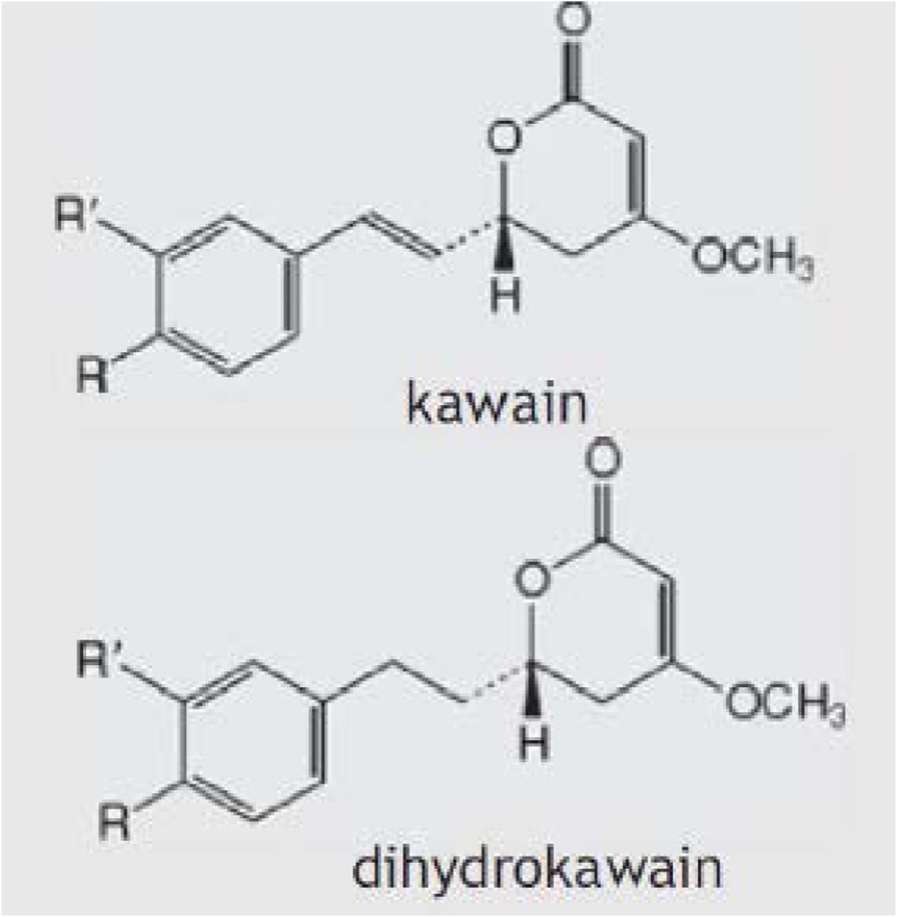
Active constituents of kava

One such option is kava *(Piper methysticum)*, a plant native to the South Pacific, whose roots have been used in traditional medicine in the form of cold-water extractions (non-alcoholic) to treat a range of health conditions, including anxiety, stress, muscular spasms, pain, and menstrual disorders [19, 20]. The therapeutic effect of kava is based on the six major lipophilic kavalactones, of which kawain and dihydrokawain (see Fig. 1) have the strongest anxiolytic activity [21]. Limbic structures of the brain have previously been suggested as the principal site of kavalactone action [22]. Kavalactones exert their anxiolytic effect through an array of neurobiological activity, primarily from modulation of gamma-aminobutyric acid (GABA) receptors via blockade of voltage-gated sodium ion channels [23, 24], reduced excitatory neurotransmitter release via blockade of calcium ion channels [25, 26], and enhanced ligand binding to GABA type A receptors [27]. Other neurochemical effects include reversible inhibition of monoamine oxidase B [28], inhibition of cyclo-oxygenase [29], and reduced neuronal reuptake of dopamine [30] and prefrontal cortex noradrenalin [31]. This noradrenergic effect differentiates the central bio-behavioural effects of kava from those of alcohol and benzodiazepines [32], while the combination of GABA modulation and increased noradrenergic activation contributes to feelings of physical relaxation with increased hedonic tone, with no deleterious effects on cognition [33].

### Background evidence

A Cochrane review and meta-analysis of seven randomised clinical trials (RCTs) using kava mono-preparations (60–280 mg kavalactones) for the treatment of generalised anxiety symptoms found a significant reduction of anxiety on the Hamilton Anxiety Rating Scale (HAM-A) for kava compared with placebo (*P* = 0.01) [34]. Similarly, a more recent pooled analysis of six studies using kava versus placebo in the treatment of anxiety symptoms found a significant effect in favour of kava on the HAM-A, with an effect size (Cohen’s *d*) of 1.1 [35]. Our previous three-week double-blind, placebo-controlled, crossover RCT (*n* = 60), using a water-soluble rootstock extract of a noble kava cultivar in chronic generalised anxiety [36], involved a preparation standardised to a dose of 250 mg kavalactones. Following a placebo run-in phase there was a reduction of −9.9 points on the HAM-A in the first kava phase versus only a −0.8 point in the first placebo phase, and a reduction in the second crossover phase from kava of −10.3 points compared to a rise of +3.3 points for placebo (Cohen’s *d* = 2.24, *P* < 0.0001).

In a subsequent six-week parallel double-blind RCT [37] involving 75 participants with diagnosed GAD (58 randomised to 120 mg daily kavalactones titrated to 240 mg for non-response), a group × time interaction was found (*P* = 0.046) for a significant reduction in HAM-A scores in favour of kava over placebo. Further, kava significantly reduced participant anxiety by −4.2, representing a moderate effect size (Cohen’s *d* = 0.63). For participants with moderate-to-severe level anxiety (as assessed on the MINI Plus diagnostic interview), the treatment effect was more pronounced (*P* = 0.020), with a larger effect size (*d* = 0.80). The effects were still significant after controlling for baseline Montgomery–Åsberg Depression Rating Scale (MADRS) depression (*P* = 0.01), baseline Beck Anxiety Inventory (BAI) anxiety (*P* = 0.05), thyroid function (*P* = 0.02), and weekly caffeine use (*P* = 0.03). Further sub-analysis of participants with pure GAD and no other DSM-IV diagnosed co-morbid anxiety disorder (panic disorder, social phobia, post-traumatic stress disorder, obsessive-compulsive disorder) revealed a significant group × time interaction (*P* = 0.020; *d* = 1.28), with a reduction of −8.5 points for kava on the HAM-A compared to −2.3 points for placebo. As part of this trial we also examined five GABA transporter polymorphisms as potential pharmacogenetic markers of kava response. We observed a significant monotonic trend for two polymorphisms in which the number of rs2601126 T-alleles (*P* = 0.021) or of rs2697153 A-alleles (*P* = 0.046) was associated with significant reductions in HAM-A scores within the kava group.

## Trial objectives

The primary objective of this trial is to confirm the effectiveness and safety of kava as a pharmacological approach to treating GAD, by conducting a longer term, multicentre RCT with a large sample using a standardised pharmaceutical-grade aqueous rootstock extract of a noble kava cultivar in participants with current diagnosed GAD. As secondary objectives, we also seek to: 1) replicate our previously observed association between genetic variation in the GABA transporter and kava treatment response, 2) elucidate the mechanisms underpinning the efficacy of kava within the GABA and glutaminergic pathways through functional and metabolic neuroimaging, and 3) examine the effects of kava on the expression of selected genes within the GABA, glutamate, dopamine, serotonin, and adrenergic pathways.

### Hypotheses

#### Primary hypothesis

1. Kava will be superior to placebo in the treatment of GAD symptoms, assessed by change in Structured Interview Guide for the Hamilton Anxiety Rating Scale (SIGH-A) [38] scores after the 16-week treatment phase.

#### Secondary hypotheses

2) Kava will be superior to placebo on a range of secondary affective disorder outcome scales (Beck Anxiety Inventory [39], Penn State Worry Questionnaire [40]*,* and Montgomery–Åsberg Depression Rating Scale [41]).
3) Kava will be superior to placebo on health-related quality of life measured using The World Health Organization Quality of Life - BREF (WHOQOL-BREF) [42], the Social Re-adjustment Rating Scale [43], and the Kessler Psychological Distress Scale [44].
4) Overall sexual satisfaction and performance rated on the Arizona Sexual Experiences Scale [45] will be in favour of kava compared to placebo, in women but not men.
5) Personality trait factors including anxiety, extraversion, socialization, and aggression rated on the Swedish Universities Scales of Personalities (SSP) may be associated with GAD symptomatology and will be measured via this self-reporting measure.
6) Anterior cingulate cortex (ACC) activation and metabolite profiles will be differentially affected in the kava treatment group over placebo from baseline to week 8. Specifically, for ACC metabolite levels, there will be an increase in GABA and decrease in glutamate/glutamine ratio (Glu/Gln); ACC activation: reduction in activation during resting state; ACC activation: reduction in activation associated with anticipatory anxiety in a task-dependent manner. Further, individual differences in rostral ACC activation measured pre-treatment may predict GAD treatment outcomes for kava compared to placebo. Increased levels of activity in the ACC will also be associated with better clinical outcome in GAD participants.
7) Response to kava will be moderated by GABA transporter polymorphisms. Specifically, for every rs2601126-T allele or rs2697153-A allele an individual carries, the greater the reduction of anxiety that will be observed.
8) Individuals in the kava group will have distinct gene expression change profiles compared to the placebo group. Specifically, we hypothesise that expression of GABA pathway genes will be up-regulated following kava treatment and remain stable in the placebo group.

## Methods/Design

### Study design and plan

**Fig. 2:**
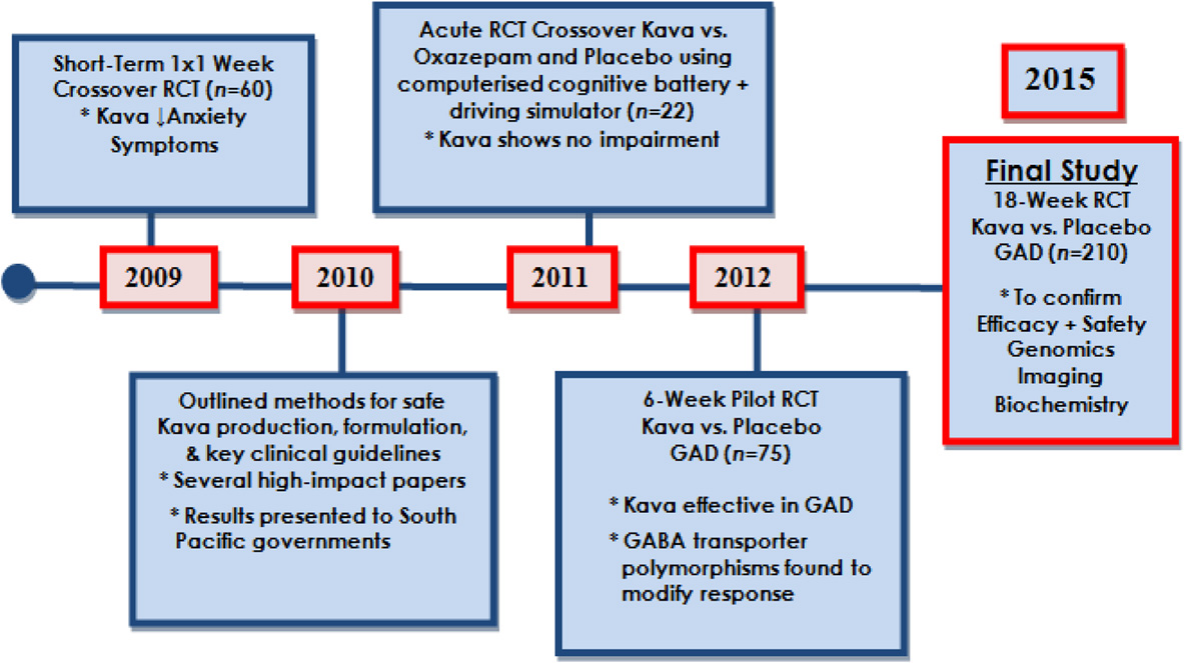
Kava studies timeline summary

The design of the study is a phase III, multi-site, two-arm, 18-week, randomised, double-blind, placebo-controlled trial using a standardised pharmaceutical-grade, water-soluble extract of kava (240 mg of kavalactones per day) or matching placebo (inert plant-based fibre) in 210 currently anxious participants with GAD. The trial sites are at the Centre for Human Psychopharmacology (Swinburne University of Technology), Hawthorn, Melbourne, Australia and the Academic Discipline of Psychiatry (The University of Queensland) based at the Royal Brisbane and Women’s Hospital, Herston, Brisbane, Australia. Recruitment is scheduled to commence September 2015 and through to June 2017 (Fig. 2).

**Fig. 3:**
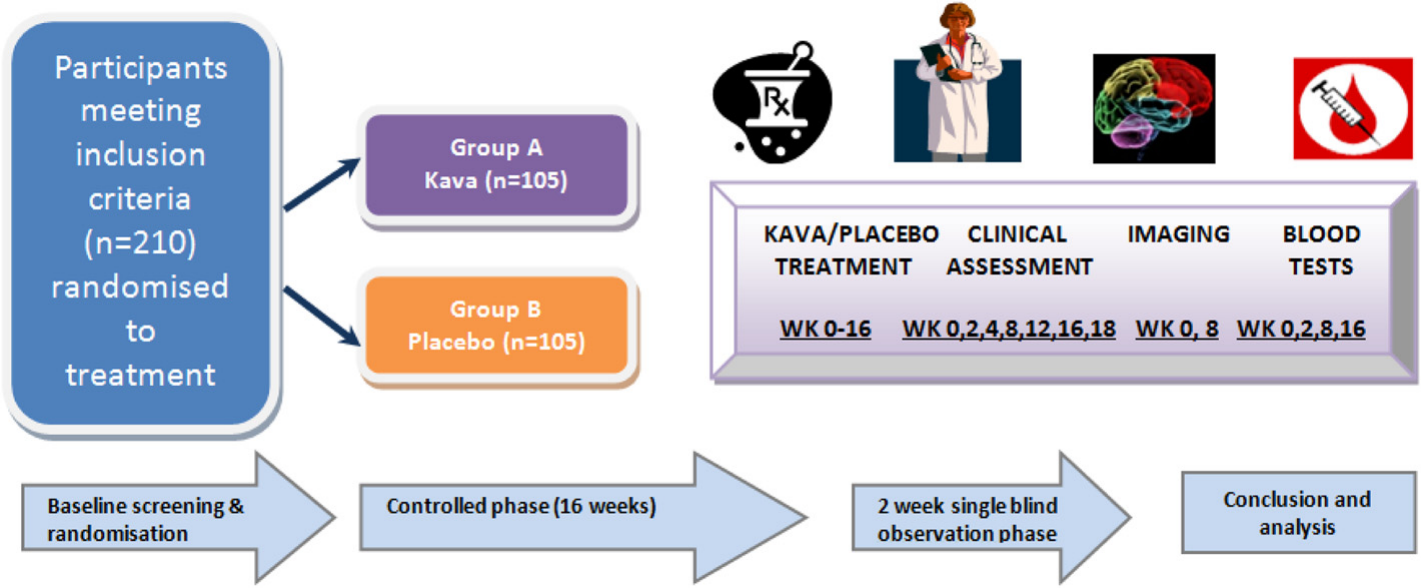
Clinical trial flowchart

Participants will be required to attend seven visits at the study sites at week 0 (baseline), 2, 4, 8, 12, 16, and 18. At the baseline visit, participants will complete informed consent forms, screening assessments, and mood and anxiety questionnaires (self-reporting and interview-based). Brain imaging component visits (Melbourne site only) are to be scheduled within one week of baseline and week 8 visits.

The study employs an additional two-week single-blind placebo-controlled post-study observation period (that is, for a total trial length of 18 weeks - refer Fig. 3).

All eligible participants will be randomly allocated to a treatment arm, and corresponding treatment will be provided following clearance of blood tests taken at the baseline visit. All subsequent visits will follow the same outline of the baseline session excluding consent forms and screening assessments and including a safety assessment. In addition, participants will be required to provide a blood sample within several days of the baseline, and at week 2, 8, and 16 visits. For female participants not taking hormone-modulating contraception who are otherwise eligible to partake in the brain imaging components, the baseline visit is to be scheduled on days 1–5 of the menstrual cycle.

### Ethics and trial registry

This study has ethical clearance (Alfred Hospital Research Ethics Committee 137/14, University of Queensland Research Ethics Committee 2014000876, Swinburne University Human Research Ethics Committee 2014/204), and is registered on ClinicalTrials.gov (protocol number: NCT02219880).

### Recruitment

Participants will be recruited through various advertisement sources:

- A dedicated study website
- Advertisements in local newspapers and radio
- A press release which may involve featured articles in newspapers and appearances by the chief investigator on radio and TV
- Online advertisements, including the Swinburne University ‘Participate in Research’ webpage (http://www.swinburne.edu.au/lss/chp/projects/kava.html) and the websites of local anxiety disorder organisations (for example, Anxiety Disorders Association of Victoria, Anxiety Recovery Centre Victoria), Google, and Facebook
- Posters and brochures displayed on campus and in the waiting rooms of local Healthscope pathology centres and medical clinics

### Primary outcome measure

The primary outcome is symptoms of GAD measured by the Structured Interview Guide for the Hamilton Anxiety Rating Scale (SIGH-A), at baseline and subsequent visits up to 18 weeks following randomization. The SIGH-A is a widely used measure of anxious symptomatology in GAD research. It includes measures of persistent worry, anxiety, mood, and somatic symptoms. The SIGH-A has been used in previous studies of pharmacological and psychological treatments for people with GAD. It is reliable (intraclass correlation coefficients for inter-rater reliability of 0.98 and test-retest reliability of 0.88), has high convergent validity with structured clinical ratings of symptoms of GAD (for example, MINI PLUS 6.0 [46]), and is sensitive to change [47, 48].

### Secondary outcome measures

- In addition, the Beck Anxiety Inventory (BAI) will be used as a participant self-report scale to assess anxiety levels. It consists of 21 items ranging from 0 (not present) to 3 (severe). These items are more somatically orientated than the SIGH-A and provide additional information on anxiety outcomes.
- The Penn State Worry Questionnaire (PSWQ) is a 16-item self-report measure of the intensity and excessiveness of worry. Examples of items are “Many situations make me worry” and “Once I start worrying, I cannot stop.” Participants rate items on a 5-point Likert scale from 1 (“Not at all typical of me”) to 5 (“Very typical of me”). Total scores range from 16 to 80, with higher scores indicating pathological worry. The PSWQ is widely used and has shown good to very good internal consistency, test-retest reliability, and discriminant validity for GAD versus other anxiety disorders [49].
- Mood will be assessed at each visit using the SIGMA (Structured Interview Guide for the Montgomery–Åsberg Depression Rating Scale (MADRS)), a diagnostic questionnaire which measures severity of depression symptoms. The SIGMA provides structured questions for each item to ensure standardisation of administration. Each item yields a score of 0 to 6, producing an overall score ranging between 0–60. A higher MADRS score indicates more severe depression. Inter-rater reliability with the SIGMA is reported to be excellent (*r* = 0.93) [50]. The use of this scale is important, as sufferers of GAD often have co-morbid depression, which may affect their response to treatment.
- Quality of life/life stressor measures will be administered at selected visits. The WHOQOL-BREF measure is a reduced version of the original instrument, designed for clinical trial use. It comprises 26 items, covering the domains of physical health, psychological health, social relationships, and environment. The Social Readjustment Rating Scale (SRRS) is a scale using ‘Life Change Units’ (LCUs) to quantify the number and severity of life stressors occurring in the preceding 12-month period. Both will be administered at baseline and at week 16. The Kessler Psychological Distress Scale (K10) is a 10-item questionnaire designed to obtain a global measure of perceived stress level, with questions pertaining to anxiety and depressive symptoms experienced in the past four-week period. For this study it will be administered at each visit.
- The Arizona Sexual Experiences Scale (ASEX) will be employed to assess the effects of kava on participants’ sexual functioning. Specifically, the self-report scale explores effects on desire, arousal level, and physiological responses over the course of the past week. It consists of five self-report items, each rated on a 6-point scale. Total scores can range between 5–30, where higher scores indicate greater sexual dysfunction. The use of this scale is important, as conventional SSRI antidepressant treatments (used to treat GAD) commonly cause sexual side effects, and results of a previous study [37] showed a significant improvement in sex drive for women. If this finding is replicated, it will show a point of difference between kava and conventional treatments. Due to the sensitive nature of the items, completion of this scale is optional.
- The Swedish Universities Scales of Personalities (SSP) is a 91-item self-rated questionnaire based on the Karolinska Scales of Personality (KSP), a scale designed to measure stable personality traits related to psychopathology [51]. It assesses four general temperament dimensions: anxiety, extraversion, socialization, and aggression. This questionnaire will be given to participants after their initial baseline visit to complete in their own time and return at the next visit.
- Genotyping of candidate and haplotype-tagging single nucleotide polymorphisms in monoamine, glutaminergic, and GABA transporter genes, measured via blood sample at week 1, will be achieved using a Sequenom Mass-Array.
- A custom RT^2^ PCR Array (Qiagen) will be used to examine gene expression of candidate genes (Table 1) at weeks 1 and 8.

**Table 1:** Gene expression list

Gene symbol	Gene name
GABBR1	GABA, B receptor 1
GABBR2	GABA, B receptor 2
GABRA1	GABA, A receptor, alpha 1
GABRA2	GABA, A receptor, alpha 2
GABRA4	GABA, A receptor, alpha 4
GABRA5	GABA, A receptor, alpha 5
GABRA6	GABA, A receptor, alpha 6
GABRB1	GABA, A receptor, beta 1
GABRB3	GABA, A receptor, beta 3
GABRD	GABA, A receptor, delta
GABRE	GABA, A receptor, epsilon
GABRG1	GABA, A receptor, gamma 1
GABRG2	GABA, A receptor, gamma 2
GABRG3	GABA, A receptor, gamma 3
GABRQ	GABA, A receptor, theta
GABRR1	GABA, A receptor, rho 1
GABRR2	GABA, A receptor, rho 2
ADCY7	Adenylate cyclase 7
ADORA1	Adenosine A1 receptor
ADORA2A	Adenosine A2a receptor
CACNA1A	Calcium channel, voltage-dependent, P/Q type, alpha 1A subunit
CACNA1B	Calcium channel, voltage-dependent, N type, alpha 1B subunit
GNAI1	G protein, alpha inhibiting activity polypeptide 1
GNAQ	G protein, q polypeptide
GPHN	Gephyrin
SNCA	Synuclein, alpha
NSF	N-ethylmaleimide-sensitive factor
P2RX7	Purinergic receptor P2X, ligand-gated ion channel, 7
SLC1A3	Glial high affinity glutamate transporter
SLC32A1	Solute carrier family 32 (GABA vesicular transporter), member 1
SLC38A1	Solute carrier family 38, member 1
SLC6A1	Solute carrier family 6 (neurotransmitter transporter), member 1
SLC6A11	Solute carrier family 6 (neurotransmitter transporter), member 11
SLC6A12	Solute carrier family 6 (neurotransmitter transporter), member 12
SLC6A13	Solute carrier family 6 (neurotransmitter transporter), member 13
ABAT	4-aminobutyrate aminotransferase
ALDH5A1	Aldehyde dehydrogenase 5 family, member A1
GAD1	Glutamate decarboxylase 1
GLS	Glutaminase
GLUL	Glutamate-ammonia ligase
PHGDH	Phosphoglycerate dehydrogenase
DRD1	Dopamine receptor D1
DRD2	Dopamine receptor D2
DRD3	Dopamine receptor D3
DRD4	Dopamine receptor D4
DRD5	Dopamine receptor D5
COMT	Catechol-O-methyltransferase
DBH	Dopamine beta-hydroxylase
DDC	Dopa decarboxylase
MAOA	Monoamine oxidase A
TH	Tyrosine hydroxylase
SLC6A3	Solute carrier family 6 (dopamine transporter), member 3
SLC6A4	Solute carrier family 6 (serotonin transporter), member 4
MAOB	Monoamine oxidase B
HTR1A	Serotonin receptor 1A
GRIA1	Glutamate receptor, ionotropic, AMPA 1
GRIA2	Glutamate receptor, ionotropic, AMPA 2
GRIA3	Glutamate receptor, ionotropic, AMPA 3
GRIA4	Glutamate receptor, ionotropic, AMPA 4
GRIK1	Glutamate receptor, ionotropic, kainate 1
GRIK2	Glutamate receptor, ionotropic, kainate 2
GRIK4	Glutamate receptor, ionotropic, kainate 4
GRIK5	Glutamate receptor, ionotropic, kainate 5
GRIN1	Glutamate receptor, ionotropic, N-methyl D-aspartate 1
GRIN2A	Glutamate receptor, ionotropic, N-methyl D-aspartate 2A
GRIN2B	Glutamate receptor, ionotropic, N-methyl D-aspartate 2B
GRIN2C	Glutamate receptor, ionotropic, N-methyl D-aspartate 2C
GRM1	Glutamate receptor, metabotropic 1
GRM2	Glutamate receptor, metabotropic 2
GRM3	Glutamate receptor, metabotropic 3
GRM4	Glutamate receptor, metabotropic 4
GRM5	Glutamate receptor, metabotropic 5
GRM6	Glutamate receptor, metabotropic 6
GRM7	Glutamate receptor, metabotropic 7
GRM8	Glutamate receptor, metabotropic 8
ADRA1A	Adrenoceptor alpha 1A
ADRA1D	Adrenoceptor alpha 1D
ADRA2A	Adrenoceptor alpha 2A
ADRB2	Adrenoceptor beta 2
ADRB3	Adrenoceptor beta 3
SLC18A2	Vesicular monoamine transporter
SLC6A2	Norepinephrine transporter
CYP3A4	Cytochrome P450 3A4
CYP2D6	Cytochrome P450 2D6
CYP2E1	Cytochrome P450 2E1
AR	Androgen receptor
NRG1	Neuregulin-1
BDNF	Brain-derived neurotrophic factor
ABCG2	Breast cancer resistance protein
ABCB1	P-glycoprotein
GAPDH	Reference genes
SADH	Reference genes
B2M	Reference genes
- Anterior cingulate cortical (ACC) region metabolite profiles, including N-acetyl-l-aspartate (NAA), creatine (Crn), glutamate/glutamine (Glu/Gln), and gamma-amino butyric acid (GABA), will be measured using single voxel magnetic resonance spectroscopy (MRS) at weeks 1 and 8.
- ACC region activity at rest and functional connectivity network (that is, default mode network) will be analysed using resting-state functional magnetic resonance imaging (fMRI) at weeks 1 and 8.
- ACC region task-dependent activation levels associated with anticipatory anxiety will be assessed using task fMRI (the International Affective Picture System (IAPS; [52]) at weeks 1 and 8.
- Acute anxiety levels related to undergoing scanner session at weeks 1 and 8 will be measured on the Spielberger State-Trait Anxiety Inventory — Trait and State components [53].

Inclusion criteria*

1. Aged between 18–70 years
2. Meets the DSM-IV and DSM-5 diagnostic criteria for generalised anxiety disorder (GAD) based on structured interview; Mini International Neuropsychiatric Interview-6.0 (MINI 6.0) [54], (note that while the MINI 6.0 uses the DSM-IV criteria, the same criteria are used in the DSM-5)
3. Presents with anxiety (SIGH-A ≥ 18) at the time of study entry
4. Fluent in spoken and written English
5. Provides a signed copy of the consent form

*MRI component – Participant eligibility for the imaging component of the study will follow a listing of restrictions typical of imaging safety at the 3-T scanner. All participants will be right-handed, with no metallic implants, piercings, or residue. For females in the imaging component, there will be conditions of enrolment relating to menstrual cycle dates, and they must not be taking hormone-modulating contraception (such as oral contraceptive pills or hormonal implants).

Exclusion criteria

1. Primary diagnosis other than GAD as determined by the MINI 6.0
2. Presentation of moderate to severe depressive symptoms (MADRS ≥ 18) at time of study entry
3. Presentation of suicidal ideation (≥3 on MADRS suicidal thoughts domain) at time of study entry
4. Current diagnosis of a psychotic disorder (for example, schizophrenia) or bipolar I on structured interview (MINI 6.0)
5. Current substance/alcohol use disorder on structured interview (MINI 6.0)
6. Currently taking an antidepressant, mood stabiliser, antipsychotic, anticonvulsant, warfarin or thyroxin, or regularly using a benzodiazepine or opioid-based analgesic (more than two days per week)
7. Current use of St John’s wort or contraindicated herbal medication
8. Previous intolerance to kava
9. Three or more failed trials of pharmacotherapy for the current GAD episode
10.  Recently commenced psychotherapy (within four weeks of study entry)
11.  Known or suspected clinically unstable systemic medical disorder
12.  Diagnosed hepato-biliary disease/inflammation
13.  Elevated liver enzymes at baseline blood test
14.  Pregnancy or breastfeeding, or trying to conceive
15.  Not using a medically approved form of contraception (including abstinence) if female and of childbearing age
16.  Unable to participate in all scheduled visits, treatment plan, tests, and other trial procedures according to the protocol.

### Treatment interventions, randomisation, and blinding

The active treatment (kava) and placebo product will be identical in colour, size, and shape, bottled and labelled with trial treatment numbers by an independent researcher. The kava is a high-quality noble *Borogu* cultivar acquired from Southern Pentecost Island, Vanuatu, and manufactured by MediHerb (Integria Healthcare (Australia) Pty. Ltd.). Each tablet is standardised to contain 60 mg of kavalactones with a higher level of kavain and dihydrokavain, and a lower relative level of dihydromethysticin. To ensure replication of odour, a small porous sachet of kava powder is inserted into all treatment bottles. The trial products will be manufactured and stored in accordance with manufacturer instructions, following Pharmaceutical Good Manufacturing Practice.

Participants are assigned study identification numbers sequentially, and treatments are randomised via identification numbers using permutated 3×2 block randomisation, for example, ABAAABABBBA, based on these numbers. Randomisation is performed by staff unrelated to the study. Treatment is allocated sequentially to enrolled participants by trial clinicians who are blinded to treatment coding (with no identifying details on the bottle of which group [A or B] each participant is assigned to, thus providing double-blinding). Participants will take two kava tablets twice per day (total of 240 mg of kavalactones per day) or matching placebo for 16 weeks.

### Statistical analyses

Analysis of data will be conducted with blinding to group allocations. The primary efficacy analysis will assess average treatment group differences for the primary outcome measure (SIGH-A) over the entire study period using a likelihood based mixed-effects model, repeated measures approach (MMRM). Results from the analysis of dichotomous data (for example, demographics and genetic data) will be presented as proportions (for example, relative risks), with 95 % confidence interval, and Fisher’s exact test *P* value where appropriate. Non-parametric statistics will be used when assumptions for parametric methods are violated. Cohen’s *d* effect sizes will be calculated. All tests of treatment effects will be conducted using a two-sided alpha level of 0.05 and 95 % confidence intervals. Data will be analysed using Statistical Package for Social Sciences software (SPSS) [55].

### Imaging analysis

#### Imaging data acquisition

A 3-tesla scanner (Siemens Tim Trio MR scanner) is utilised for the neuroimaging component of the study. *Structural MRI:* A high-resolution (1×1×1 mm) T1 weighted scan will be performed. *MRS:* To measure the level of metabolite concentrations within the region of interest (anterior cingulate cortex (ACC)), single voxel MRS will be applied. PRESS sequence (TE = 30, TR = 2000, Ave = 128, weak water suppression) is first conducted to measure the main metabolism, followed by MEGA-PRESS (TE = 68, TR = 2000, suppression frequency = 1.95 ppm, Ave = 64) [54, 56–58] sequence at the identical location to measure concentration of GABA. Finally a water-unsuppressed sequence (16 averages) will be conducted for quantification. *Functional MRI:* Resting state fMRI (EPI sequence, TE/TR = 30/2500, 3×3×3 mm, 33 slices, 10-min scan acquisitions) will be conducted with the subject’s eyes closed in addition to task-based cognitive activation fMRI study utilising IAPS to elicit anxiety (EPI sequence, TR = 2000, 3×3×3 mm, 27 mins).

#### Image data analysis

*MRS*: LC-model will be used to fit and quantify the concentration of all metabolites (total N-acetyl-l-aspartate, creatine, total choline, myo-inositol, glutamate/glutamine, and gamma-aminobutyric acid (GABA)), after quality control (that is, CLRB < 20 %, SNR >10). Further, partial volume effect will be corrected using high-resolution T1 structural image and in-house voxel co-registration script. Imaging data analysis will be conducted using Statistical Parametric Mapping (SPM) and MATLAB software [59]. *Functional MRI:* Pre-processing and statistical analysis will be conducted using SPM12 (Wellcome Department of Neurology) and associated toolboxes run on MATLAB [59]. Pre-processing will include slice timing correction, motion correction, co-registration of function and structural data, then non-linear warping into standardised stereotactic space (MNI) and spatial smoothing. Resting-state fMRI will be further band-pass filtered and de-trended, then subjected to correlational connectivity analysis using REST v1.8 (http://www.restfmri.net/forum/REST_V1.8). The pre-processed task-based fMRI data will be high-pass filter analysed using the general linear model approach in SPM12, from which contrast images of crucial interest will be generated and entered into second level group models. To test the longitudinal effect of treatment group, a flexible factorial design will be used with main effect of time and group, interaction of time × group and covariance. Significant clusters surviving multiple comparison corrections will be reported.

### Power analysis

The study will recruit a sample size of 210 participants (105 participants per arm). The study is powered to detect a small-to-moderate difference between active treatment and placebo on efficacy outcomes. Data will be analysed using data with at least one post-baseline, and all data including baseline measurement, in an intention-to-treat analysis. As such, a conservative effect size F of 0.15 on the primary outcome measure (SIGH-A) for a two-tailed analysis (with alpha = 0.05), and the study powered at 80 % (Z beta = 0.80), with a correlation among repeated measures (analysis of variance (ANOVA) model) over six time points, will require 206 participants (critical F of 3.88). A sample size of 206 (rounded up to 210) will be sufficiently powered to provide a statistical difference between active treatment and placebo groups (using the intention-to-treat analysis) with the data.

The sample size of the imaging component of this study is 40 per treatment arm (*n* = 80). Following 12-week supplement administration, a recent MRS study with a sample size of 24 (12 in each arm) detected significant changes in metabolites by 39 % [60]. Our sample size of 40 participants in each arm will provide higher statistical power compared to previous studies, whilst still maintaining sufficient power with a 10 % participant attrition rate, as well as attrition due to unreliable metabolite estimates in one or both time points.

### Safety considerations

While kava is a Therapeutic Goods Association (TGA) listed, over-the-counter nutraceutical product in Australia and has not been shown to cause any confirmed serious adverse reactions in this country, concerns over hepatotoxicity have led to withdrawal or restriction in some other countries. As discussed in the literature [61–63], many reported cases have involved concomitant ingestion of other compounds with potential hepatotoxicity (such as other medications and/or alcohol). At the clinical level, a variety of case study data from patients with kava hepatotoxicity have been gathered, with probable causation reported in a few cases [63]. Previously reported hepatotoxicity issues identified with German kava products may also be due in part to the extraction (ethanol or acetone extraction) method; the use of non-water soluble chemical (ethanol or acetone) extraction techniques (the traditional solvent is water), and use of aerial parts of the plant and root and stem peelings, and poorly prepared, potentially contaminated raw material [63].

In response to safety concerns, the World Health Organization commissioned a report assessing the risk of kava products [64]. Recommendation 2.1.3 from the report suggested that products from water-based suspension preparations be preferentially used over acetonic and ethanolic extracts. The current study addresses these safety concerns by using standardised aqueous formulations of kava from the peeled rootstock of a noble cultivar (such cultivars are higher in kawain and dihydrokavain and lower in dihydromethysticin). Note that in June 2014, the German Federal Institute for Drugs and Medical Devices (Bundesinstitut für Arzneimittel und Medizinprodukte) overturned the previous 2002 ban on the use of kava products. As a European Union regulatory and guideline body, such a change has positive ramifications for the potential reinstatement of kava products based on ongoing empirical evidence from clinical trial research.

### Monitoring and treatment compliance

Study staff involved in all aspects of participant recruitment, assessment, and monitoring hold postgraduate psychology qualifications, undergo training including inter-rater reliability of interviewer scale measures, and are supervised by psychiatrists and medical staff. This study has comprehensive medical supervision. Regardless, the occurrence of an adverse event (AE) may come to the attention of study personnel during study visits and interviews of a study subject presenting for medical care, or upon review of subject data by a study monitor. Any medical condition that is present at the time that the subject is screened for inclusion will not be reported as an AE. However, if it deteriorates at any time during the study, it will be recorded as an AE. All AEs will be graded for severity and relationship to study product. Participants will also be required to complete the Systematic Assessment for Treatment of Emergent Effects (SAFTEE) [65] at each assessment session to assess for AEs. All AEs will be recorded in the adverse event log in the participant case report form (CRF), including the seriousness, severity, and relationship to study product, duration, and outcome. In all cases, researchers will maintain contact with participants who experience an AE until it has been resolved and symptoms disappear. They will also be asked to notify their general practitioner. If a worsening of a participant’s mental state occurs, the study psychiatrist and/or clinical psychologist will be consulted and appropriate steps taken.

Liver function will be assessed pre-treatment, and participants with any abnormal results will be excluded as a safety precaution. In people with healthy liver function, it is not expected that any elevation of serum liver enzyme levels will occur (based on our previous studies using similar standardised pharmaceutical-grade extract of kava). Nevertheless, liver function will be closely monitored via blood samples at regular intervals throughout the study (weeks 2, 8, and 16).

Participants will be required to take the tablets as instructed during the 18 weeks of the study. At each assessment session they will be asked to return all unused tablets, which will be counted for compliance rate (tablet counts will be employed by an independent researcher).

### Data and documentation

Source documents will be kept in the participant files, which will be stored in a locked cabinet in a locked room accessible only to the investigators. Data from study measures will be entered into a password-protected electronic database on a secure network drive and backed up onto a USB/external hard drive stored alongside the participant files in a locked cabinet. All electronic data will be verifiable against source documents. As per Good Clinical Practice guidelines, upon request of regulatory authorities, the principle investigator will make all requested trial-related records, including source documents, available for direct access. The study files and all source data will be retained for 15 years from the date of publication of study results, in accordance with university policy. Study protocol and processes have been developed in line with Standard Protocol Items: Recommendations for Interventional Trials (SPIRIT) guidelines [66].

## Discussion

As detailed above in the literature, and through research related to the current project, there is evidence for kava’s anxiolytic effect in both subthreshold generalised anxiety and GAD. Due to WHO recommendations to test water-soluble extracts, and the importance of using high-quality rootstock extracts from noble cultivars of kava, further research is required. Although our pilot data are promising, confidence in the use of a water-soluble extract of kava for the treatment of GAD cannot be established without a confirmatory larger, longer term rigorous study. Kava for use in anxiety has many advantages including clinical evidence for efficacy and safety, a relatively low cost, and the general appeal of nutraceutical approaches in the provision of treatment options outside of the conventional medication armamentarium.

Further, examining the neurobiological actions that underpin the anxiolytic effects of kava may contribute to understanding anxiolytic pharmacodynamics within relevant neural pathways, in turn, better informing their use within anxiety disorders. SNPs account for the pharmacodynamics and pharmacokinetics of drug response, and the current study aims to examine the genetic profiles of study participants, and subsequent metabolic and substrate pathway functionality behind differential response to study treatment. Relevant SNPs involved in pathways such as GABA may serve as predictive markers for determining the effectiveness of kava via allelic group in the GAD sample.

The pharmacogenomic component of the study is to be integrated with an investigation of brain metabolites and neurobiological function underpinning response to kava administration.

The interface between the purported anxiolytic properties of kavalactones, the functional and metabolic properties of brain regions associated with the disorder, and the differential response in GAD symptomatology (guided by pharmacogenomic markers) is of particular interest. Several studies have examined kava modulation on neurotransmitters *in vitro* (for a review see [67]), yet to date no studies have examined *in vivo* mechanisms of action which may underlie cognitive and physiological effects of kava prescription. Changes to the properties of GABA metabolites as a product of the intervention may be measurable in limbic brain regions in the GAD sample via MRS [54, 60, 67–70] and could be the key to understanding the neurobiological efficacy of kava administration.

The anterior cingulate cortex’s (ACC’s) connectivity from lower limbic and hippocampal regions to the prefrontal cortical regions implies a central role in the organisation of affective and cognitive information. The ACC is thought to mediate affective and cognitive aspects of phenomena such as sadness recall, selective attention, loss, error and reward, suppression of negative emotion or cognitions, and autonomic processes that underpin anxiety states [71–79]. As such, (dysfunction within) the ACC may be a principal region underpinning GAD symptomatology, and is therefore the specific region under investigation for changes related to kava ingestion in the current study.

Some limitations to the study design are recognised. First, the design does not include a positive control product such as an SSRI, which may on some measures outperform the active treatment. Second, the use of imaging and genomic testing will need to ensure statistical correction applied for multiple comparisons, and thus the sample size must be reached to ensure adequate power in order to confirm any findings. Third, consideration of the external generalisability of study findings must be given, as the study sample will be ‘pure GAD’, that is, GAD that is currently untreated without co-morbid conditions such as major depression, and with no current symptom treatment regime, either pharmaceutical or psychotherapeutic.

In summary, if this traditional extract of kava is confirmed as safe and effective, it will provide a significant ‘Level 1’ treatment option which may help sufferers of anxiety and provide significant support to use in a clinical setting. It may also ease concerns about the potential reinstitution to restricted markets, which would provide socioeconomic benefit to poorer Pacific Island nations.

## Trial status

Recruitment to commence September 2015.

## Abbreviations

ACC: anterior cingulate cortex
AE: adverse event
ANOVA: analysis of variance
ASEX: Arizona Sexual Experiences Scale
BAI: Beck Anxiety Inventory
CRF: case report form
DSM-IV: Diagnostic and Statistical Manual of Mental Disorders - Fourth Edition
GABA: gamma-aminobutyric acid
GAD: generalised anxiety disorder
HAM-A: Hamilton Anxiety Rating Scale
IAPS: International Affective Picture System
MADRS: Montgomery–Åsberg Depression Rating Scale
MINI-6.0: Mini International Neuropsychiatric Interview Version 6.0
MMRM: Mixed-effects model, repeated measures
MRS: magnetic resonance spectroscopy
NAA: N-acetyl-l-aspartate
NHMRC: National Health Medical Research Council
PSWQ: Penn State Worry Questionnaire
RCT: randomised clinical trial
SAFTEE: Systematic Assessment for Treatment of Emergent Effects
SIGH-A: Structured Interview Guide for the Hamilton Anxiety Rating Scale
SIGMA: Structured Interview Guide for the Montgomery–Åsberg Depression Rating Scale
SNP: single nucleotide polymorphism
SPSS: Statistical Package for Social Sciences software
SRRS: Social Readjustment Rating Scale
WHOQOL-BREF: World Health Organization Quality of Life - BREF
